# Psychological challenges and related factors of ordinary residents after “7.20” heavy rainstorm disaster in Zhengzhou: a cross-sectional survey and study

**DOI:** 10.1186/s40359-023-01038-0

**Published:** 2023-01-06

**Authors:** Zhifeng Wang, Bing Jiang, Xingtong Wang, Dongxu Wang, Haihong Xue

**Affiliations:** 1 Department of Physical Education, Xi’an Polytechnic University, Shanxi, 710048 China; 2 Department of General Education, Shandong First Medical University & Shandong Academy of Medical Science, Tai’an, 271000 Shandong China; 3 Department of Physical Education, Xinyang University, Xinyang, 464000 Henan China

**Keywords:** Rainstorm disaster, Ordinary residents, Post-traumatic stress disorder, Anxiety, Depression, Stress, Related factors

## Abstract

**Background:**

In 2021, a once-in-a-century heavy rainstorm suddenly attacked Zhengzhou, an important inland city in northern China. However, there have been no studies on the psychological health of disaster-stricken residents. This study is the first to comprehensively report on the mental health status and related factors of local ordinary residents after the heavy rainstorm.

**Objective:**

The purpose of this study is to investigate the mental health status and related influencing factors of local ordinary residents after the flood disaster, and to provide reference for government departments to formulate disaster psychological intervention countermeasures based on evidence-driven strategies.

**Methods:**

The snowball sampling technique was used in this study, and measurement tools of Rainstorm Exposure Questionnaire, Subjective Perception of Rainstorm, Post-Traumatic Stress Disorder Checklist-Civilian version (PCL-C), Depression, Anxiety and Stress Scale-21 (DAS-21) and Chinese version of Social Support Rating Scale (SSRS) were used to evaluate the rainstorm exposure, subjective perception of the rainstorm, psychological symptoms and social support of the disaster-stricken residents within a week after the rainstorm. Logistic regression analysis was used to examine the psychological status and related factors of local residents after the rainstorm disaster.

**Results:**

A total of 469 valid samples were obtained in this study. All the subjects were in the disaster area and experienced the rainstorm personally, with normal intelligence. The statistical results showed that 25.37% people had experienced at least three rainstorm-related stresses, nearly 20.26% people had post-traumatic stress disorder (PTSD) symptoms, and 39.3%, 53.92% and 65.83% people had depression, anxiety and stress symptoms, respectively. Multivariable logistic regression analyses indicated that female (all *p* < 0.05), the divorced, agricultural workers/farmers (all *p* < 0.05), students (all *p* < 0.05), people experiencing at least three rainstorm-related stresses (*p* < 0.05 or *p* < 0.01), people with lower satisfaction at the social flood fighting measures (*p* < 0.05 or *p* < 0.01) and people with low social support (*p* < 0.05 or *p* < 0.01) were all independent risk factors for poor psychological health, and college education or above (*p* < 0.05 or *p* < 0.01), the lower degree of worrying about themselves (all *p* < 0.01), family members (all *p* < 0.01) and family property (all *p* < 0.01) were all related to higher psychological health among flood survivors after the disaster.

**Conclusions:**

Rainstorm could cause local residents to have various degrees of psychological symptoms. This study identified factors associated with the psychological health of disaster-stricken residents, which could be used to develop psychological interventions in improving psychological health of local residents.

## Introduction

In recent years, with global warming, various natural disasters such as floods, droughts, hurricanes, tsunamis and so on have occurred more and more frequently around the world [1]. All kinds of natural disasters not only caused lots of property losses and casualties, but also had a severe and lasting impact on people’s psychological health [2, 3]. Studies have found that physiological and psychological disorders after natural disasters include anxiety, post-traumatic stress disorder (PTSD), depression [4–7], fear, night terror, aggression, inattention, sleep disorders [8], extreme shyness and enuresis in children [9]. Moreover, the adverse effects of natural disasters on people’s psychological health would not disappear automatically with the end of the disaster. On the contrary, such adverse effects would last for a long time. For example, five months after the major flood disaster in Bangladesh in 1988, adolescent aggressive behavior and enuresis patients in the disaster area still increased by 10% and 34% compared with that before the flood [9]. It was also reported that 17 years after the 1998 Dongting Lake flood in China, 9.5% and 9.2% of the residents still suffered from PTSD and anxiety [10]. Kraemer et al. also reported that two and a half years after the 2004 Indian Ocean tsunami, nearly 16.8% and 17.8% of Swiss tourists affected by the tsunami still suffered from PTSD and anxiety [11]. It was also showed that one year after the flood caused by Hurricane Katrina in the United States in 2005, psychological health problems of disaster-stricken residents were still severe, with a 50% increase in mortality, and high-risk behaviors such as PTSD, suicide, serious psychological illness and drug abuse were also significantly higher than before [12]; 3–6 months after the 2007 summer floods in the UK, 22% and 48% of residents, respectively, still had serious PTSD and anxiety [7]. The long-term adverse effects of natural disasters on psychological health not only reduced the quality of life of disaster-stricken residents and severely impaired their cognitive and social function, but also acted as important risk factors, causing suicide and various psychological diseases [13, 14]. Therefore, it is of great practical significance to comprehensively evaluate the psychological health status of disaster-stricken residents and explore the relevant risk factors for formulating prevention and intervention countermeasures of psychological crisis.

Zhengzhou is the capital of Henan Province and an important inland city in northern China, with resident population of 12.6 million. It has warm continental climate with average annual rainfall of about 640.9 mm. Heavy rainfall began continuously in Zhengzhou on July 17, 2021, and it evolved into heavy rainstorm on July 20. From 18:00 on July 19 to 18:00 on July 20, the cumulative rainfall reached 505.6 mm, creating the highest rainfall per day in the city's history. At 17:00 on July 20, the flood control response was upgraded from level II to level I. Flood disaster caused by heavy rainstorm resulted in severe casualties and property losses. As of August 2, a total of 14.5316 million people in 150 Henan counties were affected, 89,001 houses were collapsed, 8.72 million acres of crops were damaged, and direct economic loss was 114.269 billion yuan. 302 people died and 50 people were missing, in which 292 people died and 47 people were missing in Zhengzhou. After Henan flood disaster, the central government of China quickly launched flood rescue measures, and other provinces and regions also provided strong assistance. However, these rescue measures mainly focused on living supplies, economy and physical illness, and paid less attention to the psychological health of disaster-stricken residents. At present, there have been no reports on the psychological health of residents after the flood disaster in Zhengzhou. In addition, although there have been a large number of reports that natural disasters damaged the psychological health of disaster-stricken residents, and even some researchers proposed valuable psychological intervention solutions, most of the studies have mainly evaluated a certain aspect of the psychological symptoms of disaster-stricken residents, there have been no studies comprehensively evaluate the subjective perception, social support, PTSD, depression, anxiety, stress and other psychological states of disaster-stricken residents by comprehensive use of multiple evaluate tools.

This study intends to use Rainstorm Exposure Questionnaire, Subjective Perception of Rainstorm, Post-Traumatic Stress Disorder Checklist-Civilian version (PCL-C), Depression, Anxiety and Stress Scale-21 (DAS-21) and Chinese version of Social Support Rating Scale (SSRS) to make a comprehensive evaluation of survivors’ subjective perception, social support, PTSD, depression, anxiety, stress and other psychological symptoms, and comprehensively analyze internal and external factors associated with individual psychological health after natural disasters from the aspects of government disaster rescue, disaster weather early warning and community organization. This study is by far the first research paper to comprehensively report the mental health status and related factors of residents after the "7.20" heavy rainstorm in Zhengzhou. In addition, the other innovation of this study is the comprehensive use of a variety of psychological assessment tools to systematically measure and evaluate the rainstorm exposure and mental health status of disaster-affected residents within one week after the disaster. This study further enriches the research achievements of disaster psychology, and also provides important reference for local government departments to develop psychological intervention countermeasures under major disasters based on evidence-driven strategies.

## Methods

### Participants and procedures

**Fig. 1:**
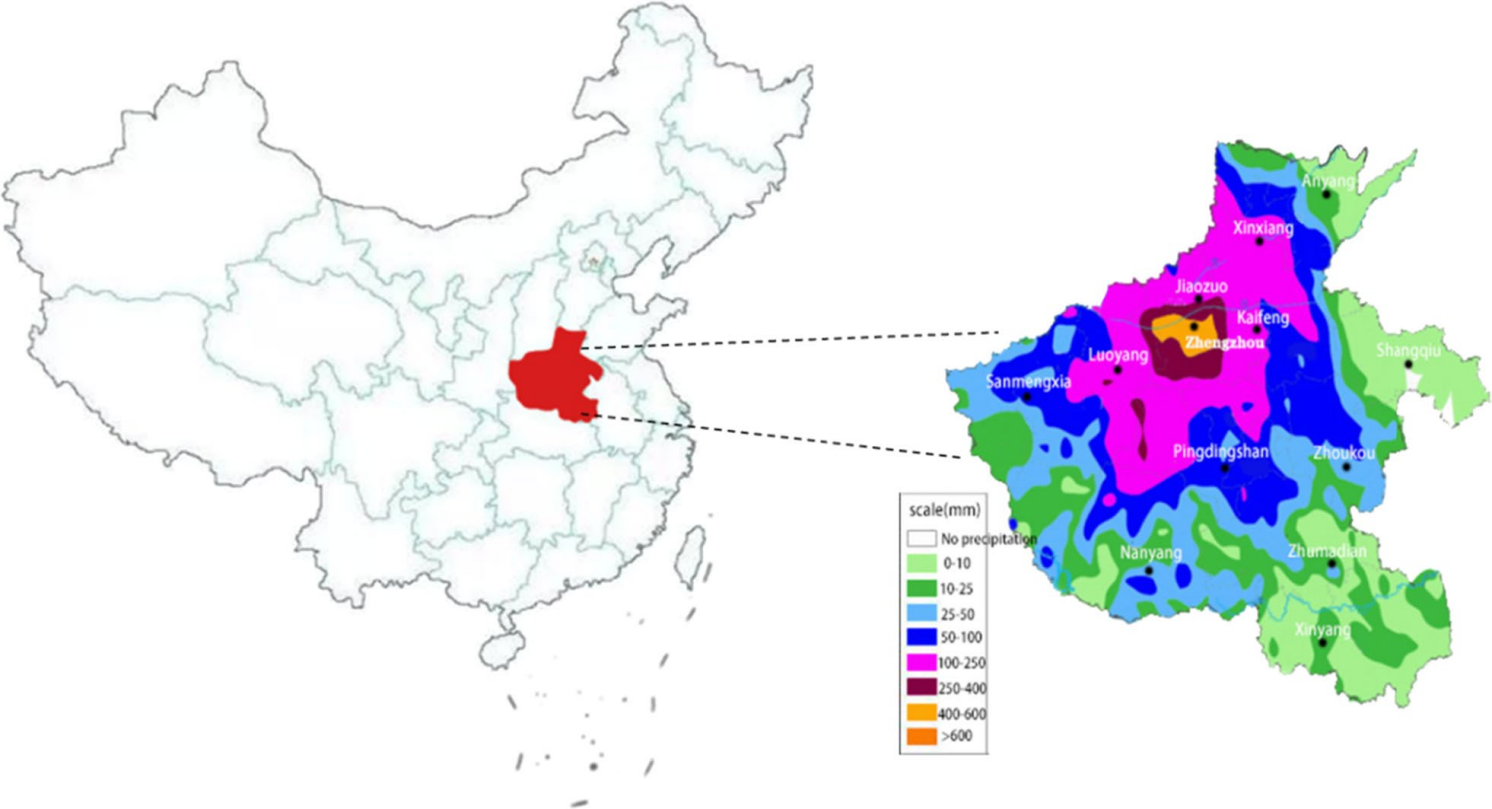
Precipitation in Henan from 8:00 on July 19th, 2021 to 18:00 on July 20th, 2021 (China Central Meteorological Observatory: http://www.cma.gov.cn/)

This study was a cross-sectional survey designed to assess various psychological symptoms and their related risk factors within a week after Zhengzhou “7.20” heavy rainstorm. In order to enable more people to participate in the survey quickly and conveniently, we adopted snowball sampling and conducted anonymous online surveys through questionnaires. A well-qualified investigator in the research group was responsible for questionnaire distribution and sample collection. This study was assisted by the person in charge of streets and communities in the disaster area, who distributed the online questionnaire to the WeChat group of the residents and encouraged more people to see the questionnaire and participate in the survey. At the beginning of the survey, all respondents were provided with informed consent information to confirm their voluntary participation in the survey. The samples were collected within one week after “7.20” rainstorm disaster, that is, from July 27, 2021 to July 31, 2021, for a total of 5 days. The inclusion criteria for valid samples were: (1) Living or working in the disaster area (Zhengzhou) from July 27, 2021 to July 31, 2021. (2) Normal intelligence, no serious mental illness. (3) The questionnaire information was basically completed without affecting data statistical analysis. Those questionnaires that did not meet the inclusion criteria were considered invalid and excluded. This study was approved by the Ethics Committee of Xi’an Polytechnic University (Approval number: 2021TY0727). Figure 1 showed the area map of single-day cumulative rainfall reported by China Central Meteorological Observatory in Henan Province from July 19th to July 20th.

### Survey instrument

#### General demographic data collection

A self-compiled questionnaire was used to investigate the gender, age, occupation, marital status and education level of the participants.

#### Rainstorm Exposure Questionnaire

According to the results on the psychological health of disaster-stricken residents after the earthquake [15, 16] and flood [10], the total amount of rainstorm-related stresses was used to evaluate the exposure of participants in the “7.20” rainstorm. Table 1 showed questionnaire of the total amount of rainstorm-related stresses. For the six questions in the tables, selecting “yes” meant the subject experienced a rainstorm-related stresses, counted 1 point; “no” meant no rainstorm-related stresses, counted 0 point, and the total score of the questionnaire was between 0 and 6. The higher the score, the more times the subject experienced heavy rainstorm-related stresses and the more severe the exposure to the rainstorm. The questionnaire was pretested in 150 samples before formal distribution, which showed that the Cronbach’s α coefficient of the questionnaire was 0.91 and split-half reliability was 0.89.

**Table 1:** Survey of the total rainstorm-related exposure

Serial number	Questions	Options
1	Have you lost at least one family member due to “7.20” heavy rainstorm?	Yes or no
2	Have you or your family members been physically injured due to “7.20” heavy rainstorm?	Yes or no
3	Have your houses been destroyed due to “7.20” heavy rainstorm?	Yes or no
4	Have you or your family lost most of your property due to “7.20” heavy rainstorm?	Yes or no
5	Have you or your family lost your livelihood due to “7.20” heavy rainstorm?	Yes or no
6	Has “7.20” heavy rainstorm seriously disrupted the normal life of you or your family?	Yes or no

#### Subjective perception of rainstorm

Because the subjective perception of "7.20" heavy rainstorm was a specific stressor after the disaster, we examined it as a stressor associated with psychological health of the residents. To evaluate the subjective perception of rainstorm, the participants were asked five questions in Table 2, and made a choice in "High", "Medium" and "Low" according to their subjective feelings. The Cronbach’s α coefficient of the questionnaire in pretest was 0.89 and split-half reliability was 0.80.

**Table 2:** Survey of the subjective perception of the rainstorm

Serial number	Questions	Options
1	Satisfaction with social flood fighting	High or medium or low
2	Satisfaction with disaster weather warning	High or medium or low
3	Worry about own life safety	High or medium or low
4	Worry about the safety of family members	High or medium or low
5	Worry about family property loss	High or medium or low

#### Social Support Questionnaire

The Chinese version of Social Support Rating Scale (SSRS) was used to evaluate social support level of participants. SSRS consisted of three dimensions: objective support, subjective support and support utilization, with 10 items in total, the score of each item ranging from 1 (none) to 4 (severe). The total score of SSRS indicated the level of individual social support. The higher the total score, the more individual social support. According to the classification standard of Feng et al. [17], the total score of SSRS at 12–44 indicated low-level social support, 45–54 was considered as moderate social support and the total score greater than 55 indicated high social support. The Cronbach’s alpha coefficient of this scale was 0.91, which had been widely used in the Chinese population and had high reliability and validity [10, 18].

#### PTSD

PTSD Checklist-Civilian version (PCL-C) was used to evaluate the level of post-traumatic stress disorder among participants. The scale consisted of 17 items and met the standard of the fourth edition of the Diagnostic and Statistical Manual (DSM-IV). It was often used as an alternative tool when structured clinical interviews could not be used [19]. The score of each item in the scale ranged from 1 (none at all) to 5 (extremely severe), and the total individual score was between 17 and 85. The higher the score, the more severe PTSD symptoms. With 38 points as the critical point to distinguish PTSD, an individual score above 38 indicated suffering from PTSD [20, 21]. In this study, traumatic events in the original PCL-C were replaced with “7.20” heavy rainstorm. Cronbach’s alpha coefficient of PCL-C was 0.96, which had been widely used in the Chinese population and showed high reliability and validity [22, 23].

#### DASS-21

Depression, anxiety and stress scale (DASS-21) was used to evaluate individual psychological health [24]. The scale consisted of 21 items, which was divided into three subscales for depression, anxiety and stress, each including 7 items. A series of 4-point Likert score was used, from 0 (not applicable to me at all) to 3 (always applicable to me). The higher the score, the more severe the emotional pain. According to literature report [25], in this study, the scores of depression subscale were divided into normal (0–9), mild depression (10–12), moderate depression (13–20) and severe depression (> 21); The scores of anxiety subscale were divided into normal (0–6), mild anxiety (7–9), moderate anxiety (10–14) and severe anxiety (> 15); The scores of stress subscale were divided into normal (0–10), mild stress (11–18), moderate stress (19–26) and severe stress (> 27). DASS-21 had been proven to be a reliable and valid measure of depression, anxiety and stress in the Chinese population [26, 27].

### Statistical analysis

Frequencies and percentages (%) were used to describe categorical data. Chi-square test was used to examine the differences in demographic variables. Variables were created to evaluate various psychological health states (PTSD, depression, anxiety and stress) of local residents after rainstorm. Univariate logistic regression analysis was conducted to determine the relationship between potentially associated variables and the psychological health states of local residents after rainstorm. Multivariate logistic regression analyses were then carried out using all significant variables obtained from the above univariate analysis as candidate variables. The 95% confidence interval (95% CI) was presented for each odds ratio (OR). All analyses were two-tailed with significant level of p < 0.05. All statistical analyses were performed using SPSS 23.0 statistical software (IBM SPSS Statistics, New York, USA).

## Results

### Participant characteristics

During one week after the questionnaire was sent out, a total of 553 people participated in the survey, including 38 questionnaires with a large number of missed items, 21 questionnaires with a large number of repeated options, and both were considered as invalid questionnaires. 25 respondents who did not provide their age or gender information were also excluded. Therefore, 469 valid questionnaires were obtained finally, yielding a response rate of 84.81% (Table 3).

**Table 3:** Characteristics of participants (n = 469)

Variable	Values	Frequency	Percent (%)	df	*p* value (/t)
Gender	Male	219	46.63	1	0.35 (χ^2^ = 0.87)
	Female	250	53.37		
< Age (years)	< 17	40	8.53	3	< 0.01 (χ^2^ = 29.09)
	18–40	206	43.92		
	41–65	176	37.57		
	≥ 66	47	10.21		
Marital status	Married	242	51.6	1	0.39 (χ^2^ = 0.92)
	Single/divorced/widowed	227	48.4		
Educational attainment	≤ Junior middle school	104	22.17	2	0.05 (χ^2^ = 4.34)
	Senior middle school	164	34.97		
	≥ University (Bachelors, Masters or Doctorate)	201	42.86		
Employment status	Unemployed/retired	34	7.25	3	< 0.01 (χ^2^ = 30.37)
	Agricultural workers/Farmers	94	20.04		
	Student	187	39.87		
	Enterprise staff	154	32.84		

*df* degrees of freedom

### Rainstorm-related exposure and subjective perception

**Fig. 2:**
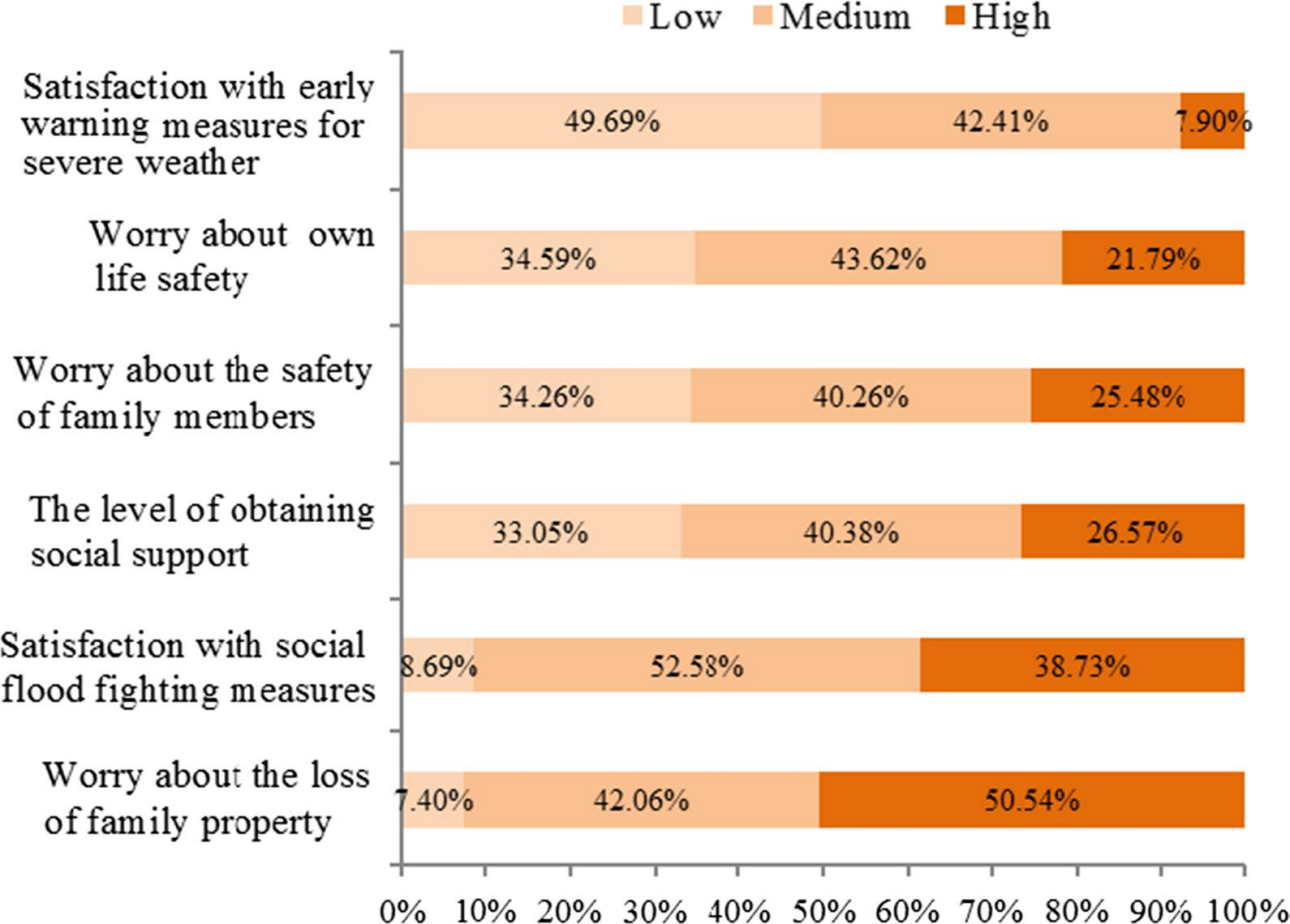
Statistics of rainstorm-related subjective perception

It was showed in Fig. 2 and Table 4 that 119 (25.37%) people experienced at least three rainstorm-related stresses, 236 (50.31%) people were satisfied with disaster weather warnings and 428 (91.31%) with social flood fighting measures, 307 (65.41%), 308 (65.74%) and 434 (92.60%) people worried about the safety of themselves, their families and family property losses respectively, moderately or highly. More than one-third of people received lower social support.

**Table 4:** Statistical table of rainstorm-related exposure and subjective perception (n = 469)

Variable	Values	Frequency	Percent (%)	df	*p* value (/t)
Rainstorm-related stressors	≤ 3	350	74.63	1	< 0.01 (χ^2^ = 24.68)
	> 3	119	25.37		
Satisfaction with social flood fighting	High	181	38.73	2	< 0.01 (χ^2^ = 38.37)
	Medium	247	52.58		
	Low	41	8.69		
Satisfaction with disaster weather warning	High	37	7.9	2	< 0.01 (χ^2^ = 44.31)
	Medium	199	42.41		
	Low	233	49.69		
Worry about own life safety	High	102	21.79	2	0.03 (χ^2^ = 6.81)
	Medium	205	43.62		
	Low	162	34.59		
Worry about the safety of family members	High	119	25.48	2	0.50 (χ^2^ = 1.27)
	Medium	189	40.26		
	Low	161	34.26		
Worry about family property loss	High	237	50.54	2	< 0.01 (χ^2^ = 38.35)
	Medium	197	42.06		
	Low	35	7.4		
Social support	High	125	26.57	2	0.51 (χ^2^ = 1.24)
	Medium	189	40.38		
	Low	155	33.05		

*df* degrees of freedom

### Prevalence of residents’ psychological health after rainstorm disaster

**Fig. 3:**
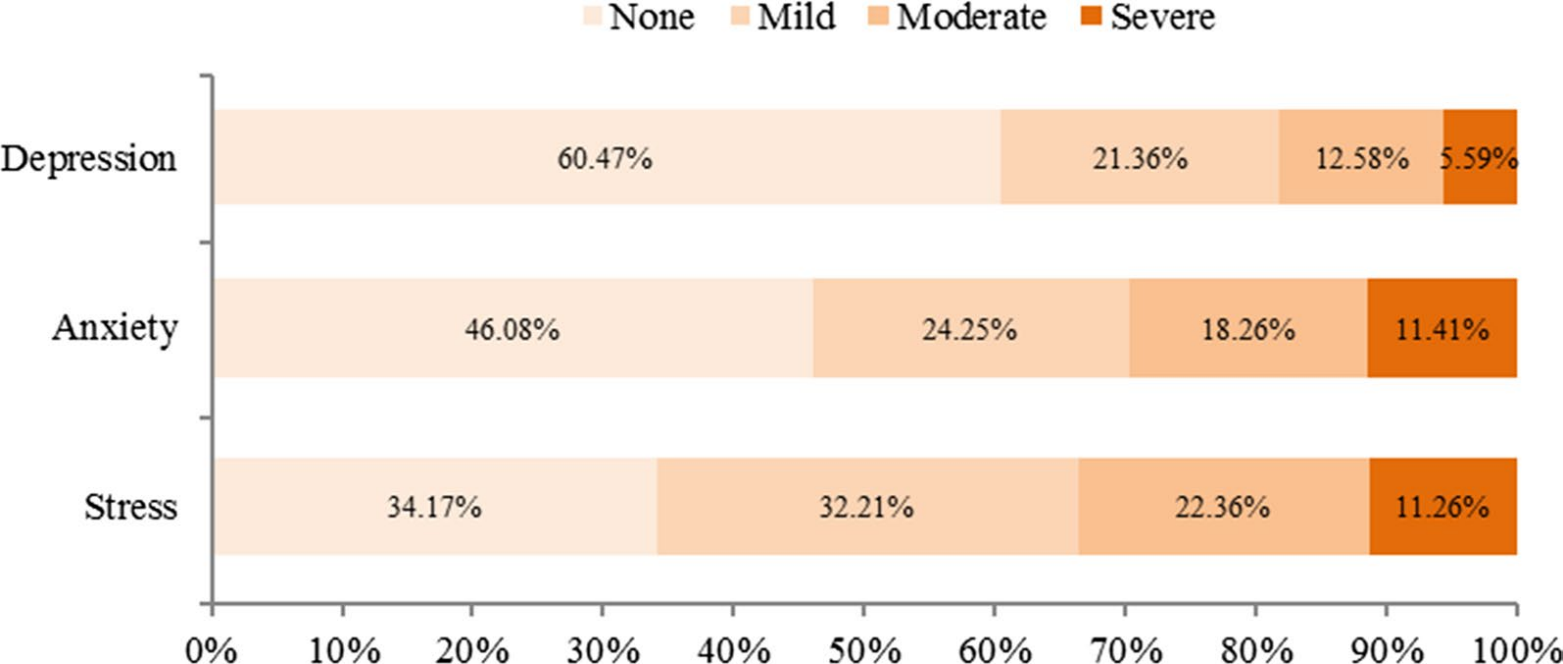
Prevalence of residents’ psychological health after rainstorm disaster

The results of DAS-21 showed that the average total score of 469 participants was 31.57 ± 8.41, of which the depression subscale score was 8.19 ± 6.77, the anxiety subscale score was 9.69 ± 7.27, and the stress subscale score was 13.47 ± 9.80. In the depression subscale, 21.36% of the participants showed mild depression symptoms, 12.58% and 5.59% had moderate or severe symptoms, respectively. In the anxiety subscale, 24.25% of the participants had mild anxiety symptoms, 18.26% and 11.41% had moderate or severe symptoms, respectively. In the stress subscale, 32.21% of the participants had mild stress symptoms, 22.36% and 11.26% had moderate or severe symptoms, respectively (Fig. 3).

The results of PTSD showed that the average total score was 32.36 ± 9.39, and 95 people (20.26%) had PTSD symptoms.

### Factors associated with psychological health

#### Univariate analysis

Univariate logistic regression analysis showed that female (R^2^ = 0.11, β = 1.77, OR = 5.87, 95% CI 2.58–13.37, *p* < 0.01), experiencing at least three rainstorm-related stresses (R^2^ = 0.15, β = 1.09, OR = 3.00, 95% CI 1.43–6.37, *p* < 0.01), lower satisfaction with social flood fighting (R^2^ = 0.11, β = 1.14, OR = 3.11, 95% CI 1.88–5.17, *p* < 0.01) and lower social support (R^2^ = 0.17, β = 1.70, OR = 5.44, 95% CI 2.78–10.65, *p* < 0.01) were all associated with higher PTSD. On the contrary, higher education (bachelors, masters or doctorate) (R^2^ = 0.09, β = − 0.68, OR = 0.51, 95% CI 0.31–0.84, *p* < 0.01), lower fear for own life (R^2^ = 0.12, β = − 0.75, OR = 0.49, 95% CI 0.42–0.94, *p* < 0.01), the safety of family members (R^2^ = 0.13, β = − 1.19, OR = 1.48, 95% CI 1.42–6.98, *p* < 0.01) and family property (R^2^ = 0.10, β = − 0.89, OR = 1.11, 95% CI 2.11–10.42, *p* < 0.01) were all associated with lower PTSD. In addition, the above variables showed similar correlations with depression, anxiety and stress. The study also found that student status (R^2^ = 0.08, β = 1.07, OR = 2.51, 95% CI 0.74–5.99, *p* < 0.05) and lower satisfaction with weather forecast (R^2^ = 0.10, β = 1.09, OR = 2.27, 95% CI 2.04–7.96, *p* < 0.05) were related to anxiety. However, this study did not find a significant correlation between marital status, age and the psychological state of the participants.

### Multivariate analysis

Multivariate logistic regression analyses were carried out using all significant variables obtained from univariate analysis as candidate variables, the results (Table 5) showed that females were significantly associated with more severe PTSD (β = 1.63, OR = 2.02, 95% CI 1.05–6.65, *p* < 0.05), depression (β = 1.74, OR = 2.98, 95% CI 1.04–7.14, *p* < 0.05) and anxiety (β = 1.37, OR = 2.40, 95% CI 1.11–5.98, *p* < 0.05). Agricultural workers/farmers were weakly associated with anxiety (β = 1.70, OR = 2.51, 95% CI 0.74–5.04, *p* < 0.05) and Stress (β = 1.87, OR = 2.43, 95% CI 1.66–6.06, *p* < 0.05). Student status were correlated with PTSD (β = 1.22, OR = 2.11, 95% CI 1.66–5.99, *p* < 0.05) and anxiety (β = 1.39, OR = 1.88, 95% CI 1.33–7.21, *p* < 0.05).

**Table 5:** Multivariable logistic regression analyses of the factors associated with psychological symptoms of ordinary residents after "7.20" heavy rainstorm disaster in Zhengzhou (n = 469)

Variables	Values	Model one (dependent variable: PTSD)					Model two (dependent variable: Depression)					Model three (dependent variable: Anxiety)					Model four (dependent variable: Stress)				
		*β*	OR	95% CI	*p*	R^2^	*β*	OR	95% CI	*p*	R^2^	*β*	OR	95% CI	*p*	R^2^	*β*	OR	95% CI	*p*	R^2^
*Gender*						0.29					0.32					0.34					0.21
	Male			1					1					1					1		
	Female	1.63	2.02	1.05–6.15	0.02		1.74	2.98	1.04–7.14	0.01		1.37	2.40	1.11–5.98	0.03		0.67	2.05	2.41–6.09	0.08	
*Educational attainment*																					
	≤ Junior middle school			1					1					1					1		
	Senior middle school	− 0.65	0.88	0.49–2.09	0.11		− 0.71	0.79	1.61–2.59	0.22		− 0.74	0.72	0.19–2.25	0.43		− 0.68	0.86	0.24–1.19	0.53	
	≥ University	− 1.20	0.41	0.19–4.99	0.02		− 1.19	0.44	0.16–3.92	0.02		− 1.29	0.49	0.10–6.34	< 0.01		− 0.57	0.64	0.55–1.71	0.30	
*Employment status*																					
	Unemployed/retired			1					1					1					1		
	Agricultural workers/farmers	0.68	1.83	0.98 ± 2.01	0.33		0.60	1.91	0.79–3.09	0.30		1.70	2.51	0.74–5.04	0.03		1.87	2.43	1.66–6.06	0.02	
	Student	1.22	2.11	1.66–5.99	0.04		1.09	1.49	1.81–3.18	0.09		1.39	1.88	1.33–7.21	0.02		1.11	1.50	1.07–4.60	0.05	
	Enterprise staff	− 0.61	0.71	0.31–1.61	0.40		− 0.71	0.67	0.31–1.76	0.22		− 0.39	0.87	0.03–1.97	0.51		− 0.73	0.95	0.04–1.02	0.90	
*Rainstorm-related stresses*																					
	≤ 3			1					1					1					1		
	> 3	3.57	7.57	1.03,10.24	< 0.01		2.15	2.67	1.22–7.01	0.02		3.18	2.87	1.30–9.98	< 0.01		2.22	2.90	1.08–8.64	0.02	
*Satisfaction with social flood fighting*																					
	High			1					1					1					1		
	Medium	0.87	1.22	1.57–3.98	0.20		0.68	1.50	1.83–3.45	0.52		0.42	1.29	1.36–2.99	0.63		0.39	1.71	1.31–2.21	0.42	
	Low	1.69	3.14	1.86–8.51	0.01		1.57	2.97	1.31–8.98	0.01		1.49	2.67	2.17–8.83	0.02		1.89	3.58	1.12–9.91	< 0.01	
*Satisfaction with disaster weather warning*																					
	High			1			N/A	N/A						1					1		
	Medium	0.42	1.38	0.36–2.68	0.60		N/A	N/A	N/A	N/A		0.49	1.85	0.62–3.35	0.31		0.50	1.42	0.69–3.26	0.54	
	Low	1.10	1.58	0.51–3.64	0.55		N/A	N/A	N/A	N/A		1.12	2.20	2.04–7.55	0.04		0.61	1.84	1.87–4.51	0.34	
*Worry about own life safety*																					
	High			1					1					1					1		
	Medium	0.62	0.78	0.12–2.39			0.57	0.78	1.21–2.08	0.512		0.57	0.77	1.67–2.51	0.51		0.68	0.79	1.34–2.54	0.51	
	Low	− 2.06	0.26	0.14–3.39	< 0.01		− 1.99	0.21	0.06–4.22	< 0.01		− 2.24	0.27	0.05–4.26	< 0.01		− 2.11	0.27	0.09–4.17	< 0.01	
*Worry about the safety of family members*																					
	High			1			N/A	N/A						1			N/A	N/A			
	Medium	− 0.60	074	0.93–3.85	0.51		N/A	N/A	N/A	N/A		− 0.48	0.77	0.33–3.33	0.51		N/A	N/A	N/A	N/A	
	Low	− 2.66	0.86	0.08–3.29	< 0.01		N/A	N/A	N/A	N/A		− 2.30	0.32	0.10–4.17	< 0.01		N/A	N/A	N/A	N/A	
*Worry about family property loss*																					
	High	N/A	N/A						1					1			N/A	N/A			
	Medium	N/A	N/A	N/A	N/A		0.72	0.82	0.76–3.17	0.52		0.70	0.87	0.24–5.01	0.57		N/A	N/A	N/A	N/A	
	Low	N/A	N/A	N/A	N/A		− 2.33	0.24	0.14–5.39	< 0.01		− 2.01	0.33	0.11–4.51	0.01		N/A	N/A	N/A	N/A	
*Social support*																					
	High			1					1					1					1		
	Medium	0.58	1.61	1.31–4.24	0.11		0.49	1.60	1.35–4.31	0.10		0.71	1.94	1.16–5.66	0.09		0.61	2.10	1.69–5.34	0.07	
	Low	1.69	3.11	1.05–9.36	0.01		1.58	3.08	2.24–7.99	0.02		1.60	2.67	2.51–9.28	0.02		1.24	3.10	1.11–12.68	< 0.01	

N/A: not applicable; University: including Bachelors, Masters or Doctorate

In addition, experiencing at least three rainstorm-related stresses were significantly associated with more severe PTSD (β = 3.57, OR = 7.57, 95% CI 1.03–10.24, *p* < 0.01), depression (β = 2.15, OR = 2.67, 95% CI 1.22–7.01, *p* < 0.05), anxiety (β = 3.18, OR = 2.87, 95% CI 1.30–9.98, *p* < 0.01) and stress (β = 2.22, OR = 2.90, 95% CI 1.08–8.64, *p* < 0.05). Another finding was that the lower the satisfaction with the social flood fighting measures, the higher the PTSD scale scores and each DAS-21 subscale scores. We also found that lower satisfaction of disaster weather warning information were significantly correlated with more severe anxiety (β = 1.12, OR = 2.20, 95% CI 2.04–7.55, *p* < 0.05). Furthermore, a low level of social support were also risk factors for PTSD (β = 1.69, OR = 3.11, 95% CI 1.05–9.36, *p* < 0.05), depression (β = 1.58, OR = 3.08, 95% CI 2.24–7.99, *p* < 0.05), anxiety (β = 1.60, OR = 2.67, 95% CI 2.51–9.28, *p* < 0.05), and stress (β = 1.24, OR = 3.10, 95% CI 1.11–12.68, *p* < 0.01).

In contrast, those with higher education (Bachelors, Masters or Doctorate) generally were protective factors for PTSD (β = − 1.20, OR = 0.41, 95% CI 0.19–4.99, *p* < 0.05), depression (β = − 1.19, OR = 0.44, 95% CI 0.16–3.92, *p* < 0.05) and anxiety (β = − 1.29, OR = 0.49, 95% CI 0.10–6.34, *p* < 0.01). Furthermore, the lower the degree of worry about their own life safety, the lower the scores of PTSD scale (β = − 2.06, OR = 0.26, 95% CI 0.14–3.39, *p* < 0.01), and also the lower scores in each DAS-21 subscale. Moreover, a low level of worry about the safety of family members was also a protective factor for PTSD (β = − 2.66, OR = 0.86, 95% CI 0.08–3.29, *p* < 0.01) and anxiety (β = − 2.30, OR = 0.32, 95% CI 0.10–4.17, *p* < 0.01). We also found that a low level of worry about the safety of family property loss were another protective factor for depression (β = − 2.33, OR = 0.24, 95% CI 0.14–5.39, *p* < 0.01) and anxiety (β = − 2.01, OR = 0.33, 95% CI 0.11–4.51, *p* < 0.05).

## Discussion

### Challenge of heavy rainstorm to psychological health of ordinary residents

Catastrophic events could cause a wide range of long-time psychological health and behavioral problems such as PTSD, depression, anxiety, tension, fear, insomnia, enuresis, aggression and inattention [2, 3, 5–7, 9, 28]. However, studies on psychological health problems in catastrophic events mostly focused on disasters such as earthquakes [4, 15], major infectious diseases [29–32], wars [33], hurricanes [34, 35] and tsunamis [36, 37]. China had been plagued by flood disaster for decades, but there were few studies on public psychological health problems in flood disasters caused by heavy rainstorms [38–40]. Dai W et al. investigated psychological health problems among survivors after the 1998 Dongting Lake flood [10], the results showed that even 17 years after the disaster, nearly one eighth survivors still had symptoms of PTSD and anxiety. However, our survey results showed that 20.26% of people had PTSD symptoms, 39.53% had depression symptoms, 53.92% had anxiety symptoms and 65.83% had different degrees of stress symptoms. The detection rates of PTSD and anxiety in our study were much higher than 9.5% and 9.2% reported by Dai W et al. [10], but they were basically consistent with the report by Paranjothy S et al. [7]. Paranjothy S et al. found that, 3 to 6 months after the 2007 summer floods in England, 22% and 48% of the people affected by the disaster had symptoms of PTSD and anxiety, respectively. Kar N et al. also reported that, 14 months after Super Cyclone Odisha, 26.9% and 12.0% of Indians still suffered from PTSD and anxiety, respectively [41]. The reasons for the inconsistency of the above results might be related to the different survey time, number of samples and the severity of disaster. It had been reported that the prevalence of PTSD and anxiety decreased over time [42], which might explain why the prevalence of PTSD, anxiety and stress among residents in our study was higher than that of similar studies with longer intervals. In general, unprecedented severe flood disaster could have an inevitable impact on psychological health of the local residents. Our study confirmed concerns about psychological health of the local residents after flood disaster. Simultaneously, our study also confirmed the correctness of the psychological stress theory proposed by Lazarus and Folkman, which believed that individuals would produce a series of emotional, behavioral and physiological stress responses after encountering huge disastrous events. Under this stress, people's neuroendocrine function would be in disorder. The hypothalamus pituitary adrenal cortex axis (HPA) would be especially over activated, resulting in abnormal increase of cortisol release. The long-term increase of cortisol would seriously impair the functions of immune system and nervous system, and then cause agitation, hallucination, insomnia, depression and other serious psychological health problems [43].

### Analysis on risk factors of psychological symptoms of ordinary residents after heavy rainstorm disaster

This study explored the important risk factors of heavy rainstorm disaster on the psychological challenges of ordinary residents through multivariable logistic regression analyses, which showed that women, agricultural workers/farmers, students, experiencing more than three flood-related stresses, low satisfaction with social flood fighting measures and low social support were all important risk factors for PTSD, depression, anxiety and stress symptoms. This finding was consistent with many previous studies [10, 44, 45], which found that females were more susceptible to certain psychiatric disorders after traumatic events, even during non-disastrous events, the proportion of females with various psychological problems was also larger than that of males [46]. The impact of major disastrous events on women's psychology was often stronger than that of men [47]. Single / divorced / widowed has generally strong psychological vulnerability. Even under normal circumstances, they had higher psychiatric and psychological symptoms such as compulsive disorder, depression, anxiety, fear and psychoticism than the married [48]. These symptoms were further exacerbated by rainstorm disaster. In addition, the work of agricultural workers in China was generally unstable, and their incomes were greatly affected by market fluctuations and climate disasters. The flood disaster caused a large number of factories and enterprises to shut down, farmland was flooded, rural houses were collapsed, the loss of agricultural workers might even more serious, and their psychological challenges were also more serious. The students were not mature enough, and their psychological response and adaptability under the disasters were weak, so they were often regarded as vulnerable population to psychological crises [49]. Recent studies had also found that students, agricultural workers and farmers were significantly related to psychological health problems such as anxiety and stress during the pandemic [25, 50], which was basically consistent with the results of our results. This study also showed that the rainstorm-related stresses experienced more than 3 times were significantly associated with more severe psychological symptoms (PTSD, anxiety, depression and stress). This finding was in line with many previous studies, which established that the degree of exposure to a disaster was among the most robust predictive factors of psychiatric disorders [51]. Hashemian F et al. also reported that compared with those exposed to low-intensity warfare, individuals exposed to both high-intensity warfare and chemical weapons were at an 18.6 times higher risk of PTSD and a 14.6 times higher risk of anxiety [52]. Experiencing rainstorm-related stresses more than 3 times in this study predicted the greater intensity of individual exposure to rainstorm disasters, the more serious the psychological impact. Low social support indicated that individuals were unable to obtain encouragement and help from the society, family members or relatives in catastrophic events, resulting in increased individual loneliness and stress, and aggravating various psychological health problems. Flood fighting measures by government departments or community and the early warning information of disaster weather by meteorological departments were an effective form of social support. The low satisfaction with the social flood fighting measures and the early warning information of disastrous weather would further aggravate psychological symptoms of the residents.

### Analysis on protective factors of psychological health of ordinary residents after heavy rainstorm disaster

The results of this study also showed that higher education (Bachelors, Masters or Doctorate), as well as lower worrying about the safety of self, family members and properties were important protective factors of psychological health of ordinary residents after flood disaster. This result differed from that of Wenjie Dai et al. [10], who did not find a relationship between the education level and psychological symptoms of flood disaster-stricken residents, but it was consistent with Assari S [53] and Erickson J et al. [54], who found that people's psychological symptoms decrease with the increase in academic qualifications. Generally, with the improvement of educational level, people's psychological adjustment and coping ability under major stress were also improved, so as to reduce the psychological burden under catastrophic events [55], which might be the reason for the mild psychological symptoms of those with higher education after rainstorm disaster. In addition, historically, major flood disasters often led to a large number of casualties and property losses, which reduced the sense of security and increased the pressure of residents in disaster area, and posed a serious challenge to their psychological health [12]. On the contrary, social support in major disasters could reduce the psychological pressure, loneliness and helplessness of the affected people, and act as a pressure buffer [56]. At the same time, it was also considered to be an important protective factor of various psychological crises [57], which had also been indirectly proved in this study. Therefore, in the event of rainstorm disaster, reducing the fear of disaster-stricken residents about their own and family life safety and family property loss, or giving basic social support in time can improve their sense of security and reduce psychological pressure, which will play an important role in maintaining psychological balance and health.

## Limitations

This study was of great significance to comprehensively reveal the psychological state and related influencing factors of residents after "7.20" catastrophic flood in Zhengzhou, but there were also some deficiencies. Firstly, this study was a cross-sectional survey with single sampling time and no longitudinal comparison, which led to the dynamic changes of the psychological health could not be observed in this study. Secondly, the voluntary nature of the survey might have led to a selection bias and the respondents might not represent entire population well. There was an oversampling of a particular population such as highly educated people, leading to selective bias in data results. Finally, the diagnosis of PTSD, depression, anxiety and stress of residents in this study was determined by self-report questionnaires rather than structured clinical interviews. This approach might overestimate or underestimate the prevalence of psychological symptoms among disaster-stricken residents. Therefore, it should be cautious when extending the results of this study to other areas. Despite the above limitations, this study provided valuable information for comprehensive understanding of the initial psychological responses of ordinary residents after flood disasters. More importantly, our findings could provide reference for formulating prevention and intervention policies for psychological crises of residents after major natural disaster.

## Conclusions

The detection rates of PTSD, depression, anxiety and stress of residents were high after flood disaster caused by “7.20” heavy rainstorm in Zhengzhou. Female, single/divorced/widowed, agricultural workers/farmers, students, those experienced rainstorm-related stresses more than 3 times and those received low social support were all important risk groups of psychological crises after natural disasters, they should attract the attention of social and government departments. The education level at college and above, lower level of worrying about themselves, family and family property were all associated with higher psychological health. The results suggested that, in the face of rainstorm disaster, measures to improve the personal safety and to reduce property losses in the disaster area were of great significance to improve or maintain psychological health. The above results all provided ideas for the formulation of psychological crises prevention and intervention countermeasures of disaster-stricken residents. Of course, the results of this study also highlighted the importance of psychological intervention for flood survivors timely and effectively, including cognitive behavioral therapy, which had been considered as an effective intervention for the treatment of PTSD and anxiety disorders.

## Data Availability

The datasets used and/or analyzed during the current study available from the corresponding author on reasonable request.

## Abbreviations

CI: Confidence interval
DAS-21: Depression, Anxiety and Stress Scale-21
DSM-IV: The fourth edition of the Diagnostic and Statistical Manual
OR: Odds ratio
PCL-C: PTSD Checklist-Civilian version
PTSD: Post-traumatic stress disorder
SSRS: Social Support Rating Scale
